# Antigen Delivery to Macrophages Using Liposomal Nanoparticles Targeting Sialoadhesin/CD169

**DOI:** 10.1371/journal.pone.0039039

**Published:** 2012-06-19

**Authors:** Weihsu C. Chen, Norihito Kawasaki, Corwin M. Nycholat, Shoufa Han, Julie Pilotte, Paul R. Crocker, James C. Paulson

**Affiliations:** 1 Departments of Chemical Physiology and Molecular Biology, The Scripps Research Institute, La Jolla, California, United States of America; 2 Wellcome Trust Biocentre, College of Life Sciences, University of Dundee, Dundee, United Kingdom; University of Helsinki, Finland

## Abstract

Sialoadhesin (Sn, Siglec-1, CD169) is a member of the sialic acid binding Ig-like lectin (siglec) family expressed on macrophages. Its macrophage specific expression makes it an attractive target for delivering antigens to tissue macrophages via Sn-mediated endocytosis. Here we describe a novel approach for delivering antigens to macrophages using liposomal nanoparticles displaying high affinity glycan ligands of Sn. The Sn-targeted liposomes selectively bind to and are internalized by Sn-expressing cells, and accumulate intracellularly over time. Our results show that ligand decorated liposomes are specific for Sn, since they are taken up by bone marrow derived macrophages that are derived from wild type but not Sn^−/−^ mice. Importantly, the Sn-targeted liposomes dramatically enhance the delivery of antigens to macrophages for presentation to and proliferation of antigen-specific T cells. Together, these data provide insights into the potential of cell-specific targeting and delivery of antigens to intracellular organelles of macrophages using Sn-ligand decorated liposomal nanoparticles.

## Introduction

Sialoadhesin (Sn, Siglec-1, CD169) is a macrophage-restricted surface receptor that recognizes sialic acid ligands and is conserved in human and mouse [1]. High levels of Sn/CD169 expression have been detected on resident macrophages and inflammatory macrophages in tissues obtained from patients with multiple sclerosis and rheumatoid arthritis [2]. Recent reports have shown that Sn/CD169 is involved in macrophage internalization of sialic acid carrying pathogens, suggesting that Sn/CD169 is an endocytic receptor [3], [4]. The fact that macrophages are professional antigen presenting cells has raised the possibility that targeting antigens to macrophages via Sn/CD169 would elicit antigen specific immune responses and improve host responses against poor immunogenic antigens [5], [6].

Using a porcine model, two recent reports have employed anti-Sn/CD169 antibodies to assess the potential of targeting antigens to Sn/CD169 expressing macrophages [5], [6]. Delputte et. al used an immunoconjugate comprising albumin linked to an anti-porcine-Sn antibody [5]. In another report, a murine anti-Sn antibody was used as the antigen to generate anti-murine Ig antibodies [6]. Both reports documented augmented immune responses and antibody production to the respective antigens relative to immunization with the antigens alone (albumin and murine IgG, respectively).

As an alternative to delivering antigens to macrophages using Sn-antibodies, we have explored the possibility of targeting antigens to macrophages using high affinity glycan ligands of Sn. To date this approach has been hampered by lack of a suitable platform that presents specific glycan ligands in a multivalent context that is also capable of carrying the antigen of choice [7]–[10]. Recently we reported the successful *in vivo* targeting of B lymphoma cells using doxorubicin-loaded liposomal nanoparticles decorated with glycan ligands of CD22, a B cell specific siglec [11]. Here we have adapted this platform for targeting antigens to Sn/CD169 expressing macrophages by encapsulating the antigen in the lumen of a liposome decorated with high affinity ligands specific for Sn. The multivalent presentation of glycan ligands of Sn/CD169 on the liposomes generates sufficient avidity to target macrophages and be efficiently endocytosed. Moreover, we show that liposome delivered antigen is efficiently presented to antigen-specific T cells. Our findings provide insights into targeting Sn/CD169 for delivery of antigen to tissue macrophages, and the potential for targeting Sn/CD169 macrophages to investigate their role as versatile antigen presenting cells in the innate and adaptive immune responses.

## Materials and Methods

### Ethics Statement

The Scripps Office for the Protection of Research Subjects, Institutional Review Board (IRB) has approved the use of blood from normal donors in this research. Human blood was obtained from The Scripps Research Institute’s Normal Blood Donor Service (NBDS).

The Scripps Research Institute, Institutional Animal Care and Use Committee (IACUC) has approved all animal protocols use in this research.

### Liposome Preparation

Lipids used in this study were purchased from Avanti Polar Lipids (Alabaster, AL) and NOF Corp (White Plains, NY). The Sn/CD169 ligand, 9-*N*-biphenylcarboxyl-NeuAcα2-3Galβ1-4GlcNAc-ethyl amine (3′-^BPC^NeuAc) was prepared as previously described [12]. The 3′-^BPC^NeuAc-pegylated lipid was prepared by coupling 3′-^BPC^NeuAcα2-3Galβ1-4GlcNAc-ethyl amine with *N*-hydroxysuccinimide (NHS)-activated pegylated lipids. Liposomes were prepared as previously described [11]. Non-targeted naked-liposomes were composed of DSPC: Cholesterol: PEG-DSPE in a 60∶35:5 molar ratio. Sn-targeted 3′-^BPC^NeuAc-liposomes substituted 3′-^BPC^NeuAc-PEG-DSPE for PEG-DSPE on a mol for mol basis. To prepare fluorescently labeled liposomes, 1 mol% of NBD-phosphoethanolamine or 0.2 mol% of Alexa Fluor647-PEG-DSPE made by NHS-coupling with amine-PEG-DSPE was added into the lipid mixture. Doxorubicin-loaded liposomes were used in the pharmacokinetics study. Remote loading of doxorubicin (Sigma) was obtained using gradients of ammonium sulfate [13]. To prepare ovalbumin-loaded liposomes, lipids were hydrated in solution containing 5 mg/ml OVA (Sigma) followed by liposome extrusion. Unbound OVA was removed from liposomes by passing the suspensions through a Sepharose CL-4B (GE Healthcare, Piscataway, NJ) column. The eluted fractions were collected in tubes (0.3 ml per tube) and determined for OVA concentration using a Bio-Rad Protein Assay kit. The loading efficiency of liposomal OVA was approximately 20∼30%. Liposomes prepared using this method had a mean particle size of 100±10 nm in diameter and were confirmed by a Malvern particle sizer instrument (Westborough, MA).

### Cell Lines

THP-1 cells over-expressing human Sn (TSn) cells were a gift from Dr. Hans Rempel and Dr. Lynn Pulliam (University of California, San Francisco) [14]. TSn cells were maintained in RPMI-1640 supplemented with 10% heat-inactivated fetal calf serum (FCS), 100 U/ml penicillin, 100 µg/ml streptomycin, 2 mM glutamine, 50 µM 2-mercaptoethanol, and 5 µg/ml Blasticidin S (Invivogen, San Diego, CA). CHO-K1, Daudi (Burkitt’s B lymphoma), BW5147 (mouse thymoma), and L929 (mouse fibroblast cell line) were obtained from ATCC and maintained in the same medium used for TSn without Blasticidin S. CHO cell lines expressing murine Sn/CD169 and human Siglec-9 and 10 were generously provided by Dr. Paul Crocker (University of Dundee, UK) and Dr. Yasuhiro Hashimoto (Fukushima Medical University, Fukushima, Japan) [15]. They were maintained in F10 medium supplemented as above with 0.5 mg/ml Geneticin (Invitrogen) instead of Blasticidin S. CHO cells expressing Siglec-F, and human CD22 and Siglec-8 were maintained as described [11], [16]. The retrovirus-packaging cell line Plat-E was obtained from Dr. Kazuo Yamamoto (The university of Tokyo, Japan) under the permission from Dr. Toshio Kitamura (The university of Tokyo, Japan) and maintained as described [17].

### Transfection of the CHO Cells with Siglec-E

Full length of Siglec-E were subcloned into into pcDNA5/FRT. The Flp-In CHO cell line (Invitrogen) were transfected with pcDNA5/FRT-Siglec-E to establish CHO cell lines stably expressing Siglec-E. Transfected cells were cultured in DMEM/F12 supplemented as above with 0.5 mg/ml Hygromycin-B (Roche Applied Science, Indianapolis, IN) instead of Blasticidin S.

### Transduction of the BW5147 Cells with Siglec-G

cDNA for Siglec-G was a generous gift from Dr. Lars Nitschke (University of Erlangen, Germany). The extracellular and transmembrane region of Siglec-G (amino acids 18–571) was subcloned into the pMXs-IRES-EGFP-Myc-CD3ζ vector, which allows the expression of the N-terminal Myc-tagged Siglec-G/CD3ζ fusion proteins on cell surface with the bicistronic expression of EGFP. Two days after transfecting Plat-E cells with pMXs-IG-Myc-Siglec-G-CD3ζ plasmids by Lipofectamin 2000 (Invitrogen), the culture supernatant was harvested and added to BW5147 cells with 8 µg/ml Polybrene (Millipore, Bedford, MA). BW5147 cells expressing EGFP were sorted to more than 95% by the FACSVantage SE (BD biosciences, San Jose, CA).

### Liposome Binding Assay

Cell lines or mouse primary cells were incubated with the fluorescently-labeled liposomes for the indicated period at 37°C. The stained cells were washed with HBSS containing 0.1% BSA, 1 mM MgSO_4_, and 1.3 mM CaCl_2_ (FACS buffer) and analyzed by flow cytometry with the exclusion of dead cells by propidium iodide staining. More than 10,000 total cell counts were acquired by FACS Caliber and LSRII (BD Bioscience) and data were analyzed by FlowJo (Tree Star, San Carlos, CA). In some experiments cells were incubated with liposomes in the mouse or human serum (MP Biomedicals, Solon, OH). For determining liposome internalization, liposome-stained TSn cells were washed with either FACS buffer or 0.133 M citric acid buffer (pH 3.3) that removes cell surface liposomes prior to FACS analysis.

### Fluorescence Microscopy

For liposome co-localization assay, CHO cells expressing mSn/CD169 were plated to a cover slip to achieve a 90% confluence and stained with fluorescent liposomes at 37°C for 1.5 hr. After removing unbound liposomes, cells were fixed with 4% paraformaldehyde and stained with biotin-conjugated anti-mouse Sn/CD169 (clone: MOMA-1) for 1 hr at 25°C. For detecting early endosomes and lysosomes, fixed cells were permeabilized with 0.05% saponin and stained with anti-EEA1 (BD Pharmingen) or anti-lysosome (clone: UH3) antibodies. After washing with buffer, cells were stained with an Alexa555 conjugated secondary antibody (Invitrogen). Finally, the specimens were mounted on a slide using mounting solution containing DAPI that stains nuclei. Images were taken using a Zeiss fluorescence microscope, an Axiocam camera (Carl Zeiss) and Axiovision 4 acquisition software. For additional information please see Methods S1.

### Generation of Mouse Bone Marrow Derived Macrophages (BMM)s

C57BL/6J mice were purchased from The Scripps Research Institute (TSRI) animal breeding facility. *Sn* ^−/−^ mice were kindly provided by Dr. Ajit Varki (UCSD, San Diego) with the permission from Dr. Paul Crocker (University of Dundee, UK). All mice were maintained in pathogen-free conditions and were used in accordance to the guidelines of the Institutional Animal Care and Use Committee. Bone marrow cells from C57BL/6J wild type and *Sn^−/−^* mice were harvested and differentiated into macrophages *in vitro* with RPMI-1640 medium containing 10% heat-inactivated FCS, 2 mM glutamine, 100 IU/ml penicillin, 100 µg/ml streptomycin, 1 mM non-essential amino acid, 1 mM sodium pyruvate, 50 µM 2-melcaptoethanol, 20 mM HEPES and either 10 ng/ml M-CSF (R&D Systems) or 10% L929 cell culture conditioned medium [18]. On day 7, IFN-α (500 IU/ml, R&D Systems) was added to the culture for 2 additional days to induce Sn/CD169 expression. To check Sn/CD169 expression on macrophages, cells were harvested and blocked with anti-mouse CD16/32 (2.4G2, BD Biosciences) prior to detecting with fluorescence conjugated anti-Sn and anti-F4/80 (BM8, Biolegend, San Diego, CA). The stained cells were washed with FACS buffer and analyzed by flow cytometry as described above.

### OT-II T Cell Proliferation Assay

OT-II TCR transgenic mice on C57BL/6J background were provided by Dr. Charles Surh (TSRI, La Jolla, CA). CD4^+^ T cells were purified from the spleen of OT-II mice using a CD4^+^ T cell negative selection magnetic column (Miltenyi Biotec, Auburn, CA) followed by labeling with carboxyfluorescein diacetate succinimidyl ester (CFDA-SE, Invitrogen). Naïve or IFN-α stimulated BMMs were incubated with OVA antigen alone, naked or 3′-^BPC^NeuAc liposomes carrying same amount of OVA (1 mg/ml) at 37°C for 1 hr. BMMs were washed thoroughly to remove unbound OVA before added to CFDA-labeled OT-II T cells at a BMMs to T cells ratio of 1∶20. After 3-day culture *in vitro*, cells were harvested and co-stained with anti-CD4 (GK1.5, Biolegend) prior to FACS analysis for T cell proliferation. Dead cells were excluded using propidium iodide staining.

### Sn Recycling Assay

Recycling properties of Sn were analyzed as previously described [19]. Briefly, CHO cells expressing mSn were incubated with unlabeled anti-Sn antibody (3D6, AbD Serotec) or isotype-matched control antibody for 60 min at 37°C to allow internalization of the bound antibody. Cells were then cooled at 4°C to stop the internalization and washed with either FACS buffer or acidic buffer as described above to strip the anti-Sn antibody from the cell surface. The live cells were then stained with anti-rat IgG-Alexa488 (Life Technologies) to detect the unlabeled anti-Sn antibody on the cell surface. To assess Sn recycling, the cells washed with acidic buffer were further incubated for 30 min at 37°C to allow recycling of the internalized unlabeled antibody back to the cell surface, or at 4°C as a control where no recycling can occur. The cells were then cooled to 4°C stained with secondary labeled anti-rat IgG antibody as described above and analyzed by flow cytometry.

### Statistical Analysis

We performed ANOVA and two-sample t-test for statistical analysis.

## Results

### Sn-targeted Liposomes are Selectively Bound to and Efficiently Internalized by Sn-expressing Cells

**Figure 1:**
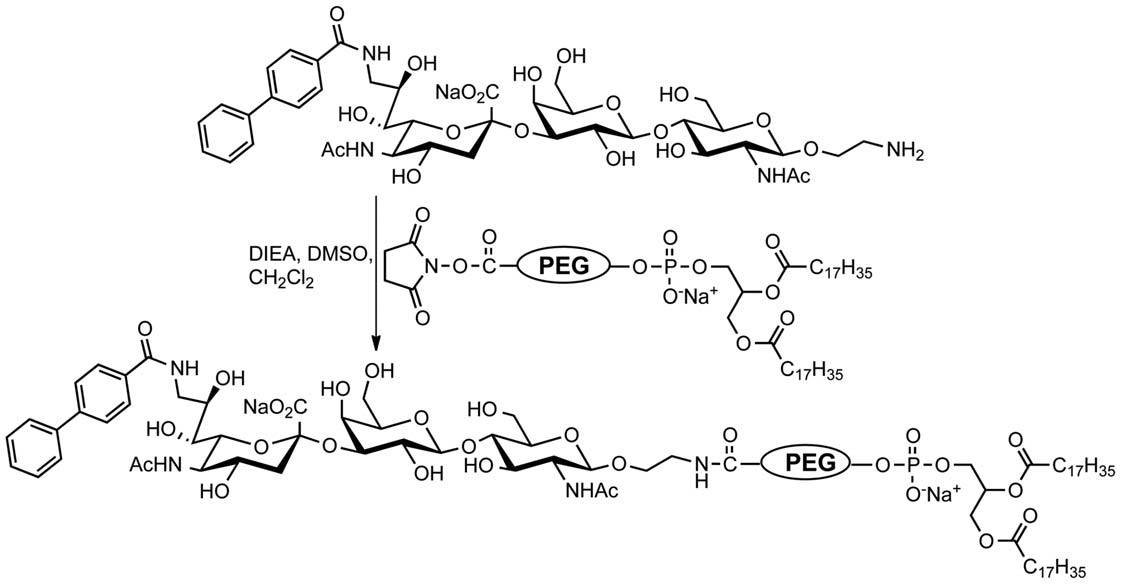
Synthesis of the 3′-^BPC^NeuAc-pegylated lipids. The sialic acid ligand with an ethylamine linker was coupled to an NHS-activated pegylated lipid.

**Figure 2:**
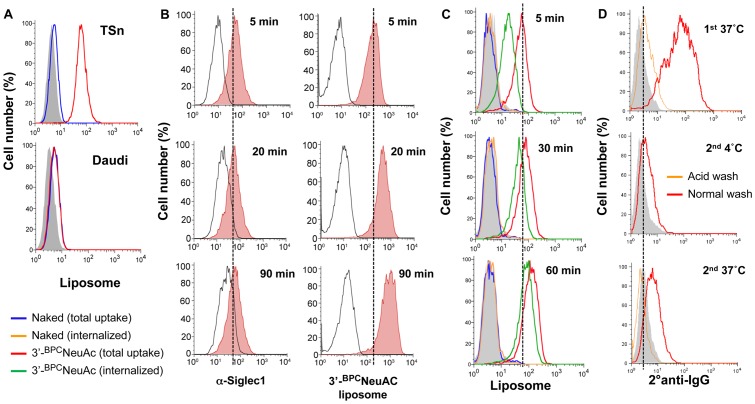
Liposomes bearing 3′-^BPC^NeuAc ligands bind to and internalized by Sn/CD169 expressing cells. (**A**) FACS analysis for binding of the naked (*blue line*) or 3′-^BPC^NeuAc (*red line*) liposomes to TSn and Daudi cells that express surface hSn and hCD22 (Siglec-2), respectively. Unstained cells (*filled grey*) were used as a negative control. Shown are results from 1 of 3 representative experiments followed indicated treatment. (**B**) Ligand-bound liposomal cargos but not antibodies exhibited time-dependent accumulation in Sn-expressing cells. TSn cells were incubated with fluorescently labeled anti-Sn antibody (Clone 7–239 (Serotech); *filled red, left panel*) or Sn-targeted liposomes (*filled red, right panel*) for 5, 20 and 90 min before they were washed with isotonic HBSS buffer prior to FACS analysis. Isotype antibody or naked liposome stained cells were used as negative controls. (**C**) Internalization of Sn-targeted liposomes by TSn cells. Cells were incubated with fluorescent naked or 3′-^BPC^NeuAc liposomes for 5, 30 or 60 min at 37°C before they were washed with isotonic HBSS buffer (pH 7) or acid buffer (pH 3.3) prior to FACS analysis to determine levels of the total liposome uptake (membrane bound plus internalized), or internalized liposomes. Cells that were not stained with liposomes were used as a negative control (*filled gray*). (**D**) Recycling of Sn between cell surface and inside the cell. *Top*; unlabeled anti-Sn Ab was incubated with Sn expressing CHO cells at 37°C. Cells were then cooled to 4°C and stained with a labeled secondary Ab after a neutral wash (*Red line*) or acid wash (*Orange line*) to detect residual cell surface bound anti-Sn Ab. Isotype control antibody staining is shown as *filled histogram*. *Middle*; acid-washed cells from the *Top* were subjected to a further incubation at 4°C and stained with labeled secondary Ab, showing that no anti-Sn Ab had returned to the cell surface. *Bottom*; acid-washed cells from the *Top* were warmed to 37°C to allow internalized anti-Sn Ab to be recycled back to the cell surface. Ab re-appearing on the surface of the cell is detected by staining the cells with labeled secondary Ab. Results shown are representative of at least two independent experiments.

Sn/CD169 has a known preference for binding to ligands bearing α2-3 linked sialic acids on glycans of glycoproteins and glycolipids. We previously identified a high-affinity glycan ligand (9-*N*-biphenylcarboxyl-NeuAcα2-3Galβ1-4GlcNAc, 3′-^BPC^NeuAc) of Sn/CD169 using a sialoside analogue array [1], [12]. To prepare Sn-targeted liposomes, we coupled 3′-^BPC^NeuAc ligands to a pegylated phospholipid (Figure 1) and the corresponding 3′-^BPC^NeuAc pegylated lipid was formulated with cholesterol and other phospholipids to make liposomes. In flow cytometric assays, fluorescently labeled 3′-^BPC^NeuAc liposomes exhibited a specific binding to Sn-expressing TSn cells and did not bind to Daudi cells that express human CD22 (Siglec-2), whereas the non-targeted naked liposomes showed no binding to either (Figure 2A). Next we compared the kinetics of binding and uptake of anti-Sn antibody and the 3′-^BPC^NeuAc liposomes at 37°C. As shown in Figure 2B, binding of both antibody and 3′-^BPC^NeuAc liposomes was evident at 5 minutes, but while the amount of bound antibody remained constant, the amount of 3′-^BPC^NeuAc liposomes increased 5–10 fold over the next 90 minutes. As documented previously for CD22 (Siglec-2) [19], these observations are consistent with release of the endocytosed liposomes in the acidic endosomes, and recycling of the ‘empty’ receptor to pick up another load of cargo at the cell surface [19], while antibody is not released, and does not accumulate. To explore this further, TSn cells were incubated with fluorescently labeled naked or 3′-^BPC^NeuAc liposomes at 37°C for the indicated time, and aliquots of cells were then briefly washed with either isotonic buffer to assess total bound liposomes, or with acidic buffer (pH 3.3) that removes surface-bound ligands, revealing the amount internalized (Figure 2C). At 5 minutes about 1/3 of the bound liposomes were internalized, but over time the percentage internalized increases such that nearly 90% is internalized by 60 minutes. The results reinforce our interpretation from Figure 2B that ligand-decorated cargo accumulates in the cell as a result of Sn/CD169 releasing the ligands in endosomal compartments, and recycling to the surface to shuttle additional cargo into the cell as documented for CD22 [19].

To test the hypothesis that anti-Sn antibody recycles to the cell surface after internalized by Sn/CD169, we conducted a recycling experiment analogous to that done to characterize recycling of CD22 [19]. We first incubated the CHO cells expressing Sn/CD169 with unlabeled anti-Sn antibody at 37°C. The cells were washed with acidic buffer to remove bound antibody on the cell surface (Figure 2D, Top). Acid-washed cells were then incubated at either 4°C as a control for no recycling (Figure 2D, Middle) or 37°C to allow recycling to occur (Figure 2D, Bottom). Cells were then stained with secondary anti-rat IgG antibody to detect the unlabeled antibody that recycled back to the cell surface from inside the cell. As seen in the bottom panel, internalized anti-Sn antibody did indeed recycle to the surface of the cell following incubation at 37°C (Figure 2D, Bottom).

### Sn-targeted Liposomes are Delivered to Endosomes and Lysozymes

**Figure 3:**
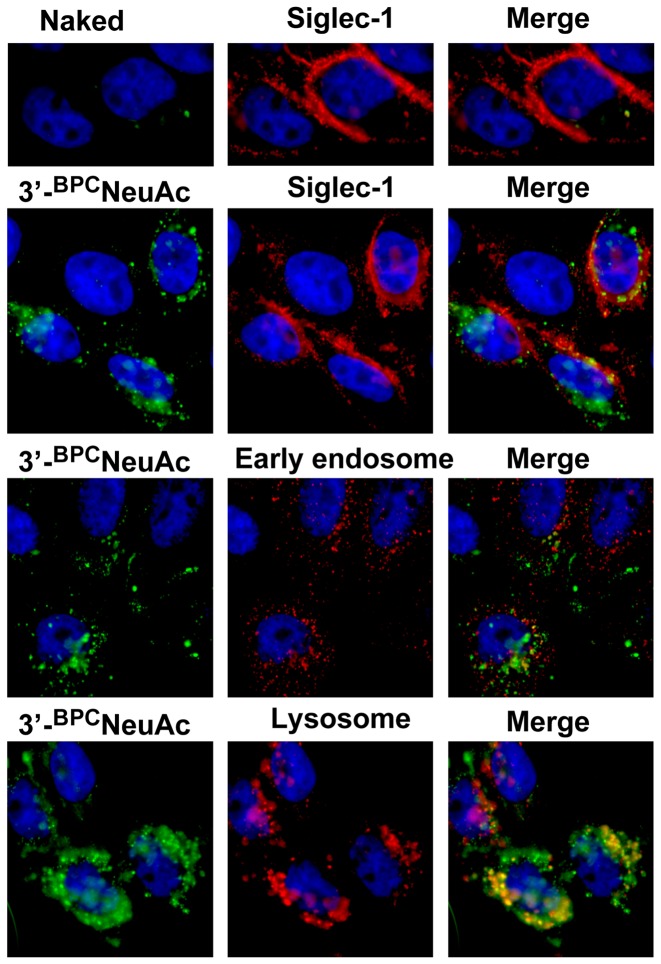
Fluorescence microscopy analysis of the endocytosis of 3′-^BPC^NeuAc liposomes in the Sn-expressing cells. CHO-mSn/CD169 cells were stained with fluorescently labeled naked or 3′-^BPC^NeuAc liposomes (*green*) followed by staining with anti-Sn (*red*) or antibodies that detect early endosomes (*red*) and lysosomes (*red*). The nuclei were visualized by staining with DAPI (*blue*).

Next we analyzed the subcellular localization of 3′-^BPC^NeuAc liposomes by fluorescence microscopic analysis using a CHO cell line expressing Sn/CD169 (Figure 3 and Figure S1). Sn-CHO cells were exposed to fluorescently labeled liposomes (green) for 90 minutes, and then co-stained with antibodies to Sn/CD169 (top panels), or to antibodies to markers of early endosomes (EEA-1) or lysosomes (LAMP1). As expected, control cells stained with non-targeted ‘naked’ liposomes show little green fluorescence. However, cells exposed to 3′-^BPC^NeuAc liposomes showed robust punctate staining of intracellular compartments. This is in marked contrast to the intense cell surface localization of the Sn/CD169 (Figures 3 and S1). The intracellular 3′-^BPC^NeuAc liposomes internalized by Sn/CD169 show modest co-localization with early endosomes, and strong co-localization with lysosomes. These data suggest that liposomes with 3′-^BPC^NeuAc ligands are efficiently endocytosed by Sn-expressing cells, and the glycan ligand-decorated liposomal cargo accumulates in lysosomes. Despite the fact that endocytosis of liposomes is mediated by Sn/CD169, there is little evidence of Sn in intracellular compartments. As discussed further below, we believe that this reflects the fact that Sn is a recycling receptor, and is predominately localized at the surface of the cell.

### Specificity of 3′-^BPC^NeuAc Liposomes for Human and Mouse Siglecs

**Figure 4:**
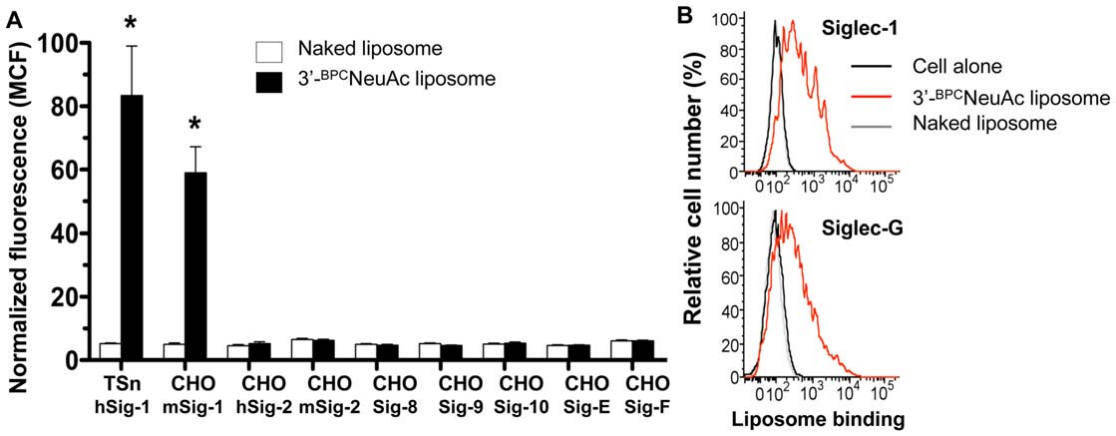
Specificity of Sn-targeted liposomes against a panel of siglec-expressing cells. FACS analysis for binding of naked or 3′-^BPC^NeuAc liposomes to siglec-expressing CHO lines and TSn and BW5147 cell lines that express hSn and Siglec-G, respectively. (A) Binding of liposomes is expressed as mean channel fluorescence (MCF) ± s.d. (n = 3). Binding degree of 3′-^BPC^NeuAc liposomes to TSn, CHO-mSn cells was statistically significant comparing to the same cell line that was treated with the naked liposomes (*P<0.05). (B) Compatible amount of 3′-^BPC^NeuAc liposome binding to CHO-mSn and SIglec-G-BW5147 cells is shown. Cells were stained with 3′-^BPC^NeuAc (*Red*), Naked liposomes (*Gray*), or unstained (*Black*). Data are representative of 2 independent experiments.

To evaluate the selectively and affinity of the 3′-^BPC^NeuAc liposomes to other siglecs, we tested binding of the naked and the 3′-^BPC^NeuAc liposomes against a panel of siglec-expressing cell lines. As shown in Figure 4A, 3′-^BPC^NeuAc liposomes exhibited strong binding to human and mouse Sn-expressing cells, and little or no binding cells expressing most other siglecs. The exception was moderate binding of 3′-^BPC^NeuAc liposomes to murine Siglec-G expressing cells, which is expressed on B cells, monocytes and dendritic cells (Figure 4B) [20]–[22]. In this regard, it is notable that they exhibited no detectable binding to cells that express Siglec-10, the human ortholog of Siglec-G. Binding of the ligand liposomes to cell lines that express other siglecs was at minimum levels comparing to that of the non-targeted naked liposomes. To assess the degree to which 3′-^BPC^NeuAc liposomes are targeted to Sn-expressing cells *in vivo*, we compared the plasma clearance rate of the naked and 3′-^BPC^NeuAc liposomes in the wild type or the *Sn^−/−^* mouse strain (Figure S2). In contrast to the naked liposomes that showed no difference in clearance between the two strains, liposomes with 3′-^BPC^NeuAc ligands were cleared approximately 8-fold more rapidly in the wild type mice than in the *Sn^−/−^* mice at 2 hr post liposome injection, suggesting that they are predominantly taken up by Sn-expressing cells in the wild type mice and remain long circulating in the mice that do not express Sn. Thus, while the 3′-^BPC^NeuAc ligand exhibits some cross-reactivity with Siglec-G, rapid clearance of the liposomes from the blood is mediated primarily by Sn/CD169 expressing cells.

### Sn-mediated Binding of Liposomes to Bone Marrow Derived Macrophages (BMM)s

**Figure 5:**
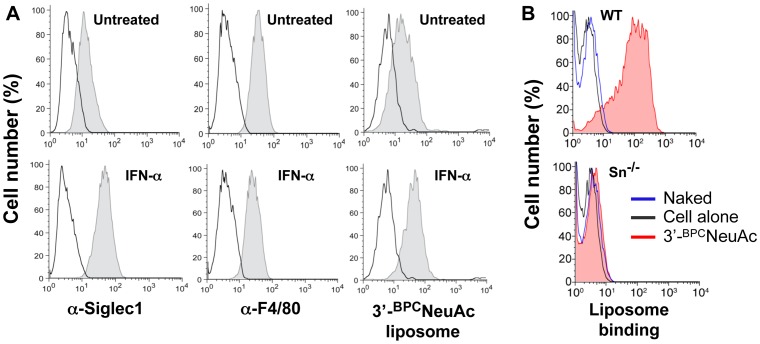
Sn-targeted liposomes bind to IFN-α stimulated bone marrow derived macrophages (BMM)s. (**A**) Mature macrophages differentiated from wild type mouse bone marrow cells were stimulated with IFN-α or left untreated before staining with anti-Sn, anti-F4/80 antibodies (*filled gray*) and Sn-targeted liposomes (*filled gray*). Cells were thoroughly washed prior to FACS analysis. Isotype antibody or naked liposome stained cells were used as negative controls (*black lines*). (**B**) BMMs derived from wild type or Sn^−/−^ mice were stained with fluorescent naked (blue lines) or Sn-targeted (*filled red*) liposomes prior to FACS analysis. Unstained cells (*black lines*) were used as a negative control. Data are representative of 3 independent experiments.

Macrophages, like dendritic cells, are antigen-presenting cells (APC) and are capable of degrading extracellular antigens into fragments and presenting digested peptides along with MHC class II molecules to CD4^+^ T cells. Since Sn/CD169 is a macrophage-specific surface receptor, we anticipate that Sn/CD169 receptor-mediated endocytosis could enhance macrophage antigen engulfment and facilitate the MHC II-restricted antigen presentation to T cells. To demonstrate the feasibility of using Sn-targeted liposomes as an efficient vehicle that could deliver antigen cargos to primary macrophages, we examined the binding of 3′-^BPC^NeuAc liposomes to macrophages that were derived from mouse bone marrow cells. Upon 7 days culture *in vitro* supplemented with M-CSF cytokine, mouse bone marrow cells differentiated into mature macrophages and expressed surface F4/80 (Figure 5A and Figure S3). These bone marrow derived macrophages (BMM)s exhibit a basal level expression of Sn/CD169 and exhibit weak binding of 3′-^BPC^NeuAc liposomes. With an additional 48-hr stimulation with IFN-α, the activated BMMs exhibited a 4–5 fold increase in surface Sn/CD169 expression, significantly enhancing the binding of the Sn-targeted liposomes (Figure 5A and Figure S3). This result is consistent with previous reports that IFN-α augmented expression of Sn/CD169 on human and porcine monocytes [14], [23]. We confirmed that binding of the liposomes to IFN-α stimulated BMMs is Sn/CD169 dependent using BMMs derived from *Sn^−/−^* strain. As shown in Figure 5B, 3′-^BPC^NeuAc liposomes bound only to BMMs derived from a wild type mouse but not to those derived from an *Sn^−/−^* strain whereas the naked liposomes do not exhibited binding to either. These results indicate that Sn-targeted liposomes bind selectively to Sn-expressing macrophages in an Sn/CD169-dependent manner.

### Sn-targeted Liposomes Deliver OVA Antigen to BMMs and Activate OVA-specific CD4^+^ T Cells

Since the internalized Sn-targeted liposomes traffic to the lysosomal compartments of the cell, where the antigen loading to appropriate MHC II molecules occurs, we investigated whether Sn-targeted liposomes are capable of delivering antigen cargo to macrophages and induce subsequent antigen presentation to T cells. For this purpose, we exploited an ovalbumin (OVA) specific T cell model. Naïve or IFN-α activated BMMs were incubated briefly with free OVA proteins or with naked or Sn-targeted liposomes that carry OVA proteins, followed by addition of purified, CFSE-labeled OT-II CD4^+^ T cells that are specific to OVA peptide 323–339 in context of MHC II I-A^b^. Since these T cells are not activated by intact OVA, T cell activation is a direct readout of the ability of the BMMs to take up and process OVA, and present the antigenic peptide on MHC II at the surface of the cell.

**Figure 6:**
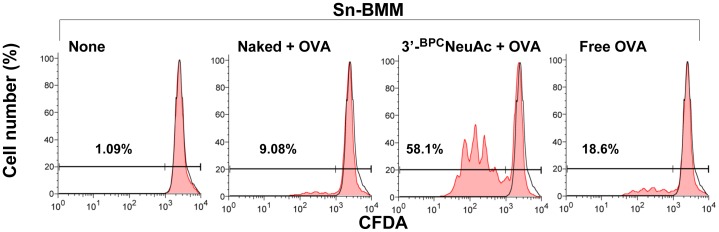
Antigen delivery to BMMs using Sn-targeted liposomes led to proliferation of antigen-specific T cells. Sn-expressing BMMs (stimulated by IFN-α) were treated with free OVA proteins, naked or Sn-targeted liposomes that carry OVA for 1 hr or left untreated as a negative control. CD4^+^ T cells purified from OT-II transgenic mice were CFDA labeled and incubated with the washed BMMs that were treated with indicated conditions. After 72 hr culture *in vitro*, T cells were harvested and analyzed for CFDA dilution by flow cytometry. Percentages of the dividing CD4^+^ T cells are indicated. Shown are representative of 2 independent experiments.

Three days after *in vitro* incubation, T cell proliferation was measured by CFSE dilution using flow cytometry. As shown in Figure 6, Sn-targeted liposomes delivered OVA efficiently to the Sn-expressing BMMs, eliciting a significant enhancement in OVA-specific T cell proliferation (58% proliferated T cells). In contrast, low levels of proliferation were seen with OVA delivered by naked liposomes or added free in solution (9.1 and 18.6% proliferation, respectively). The fact that the Sn-expressing BMMs treated with the OVA-loaded naked liposomes induced a lower level of T cell proliferation than those pulsed with an equal amount of free OVA proteins, is likely due to a shielding effect of the naked liposomes, preventing other means of uptake such as the recognition of the high mannose glycans by the mannose receptor [24], [25]. Similar experiments conducted with naive BMMs that express low levels of Sn/CD169 (Figure 5) showed no significant enhancement of T cell activation using OVA loaded 3′-^BPC^NeuAc-liposomes relative to the non-targeted naked liposomes (data not shown). The results reveal an enhanced proliferation of OVA-specific T cells by the Sn-targeted liposomal OVA relative the non-targeted formulations. These observations support our hypothesis that liposomes with glycan ligands actively deliver antigens to macrophages through Sn-mediated endocytosis, consequently enhancing the activation of antigen-specific T cells.

## Discussion

Sn/CD169 is a member of the siglec receptor family whose members are widely expressed on immune cells of man and mouse, including neutrophils, eosinophils, basophils, monocytes, B cells, NK cells, macrophages and dendritic cells [7], [8], [26]–[28]. The restricted expression patterns of siglecs on different cell types and their roles as regulators of immune cell functions, have made them attractive targets for developing cell-directed therapies [9]. Indeed, many antibody and immunotoxin based therapeutics that target siglecs are being developed as a result of their potential for cell-depletion therapies that treat leukocyte-mediated allergy, autoimmune, infectious diseases and neoplasias [5], [29]–[31].

Several reports have demonstrated an alternative approach of siglec targeting using multivalent displays of their glycan ligands. Successful examples include targeting B cells with high affinity glycan ligands of CD22 (Siglec-2) which results in an effective depletion of B cell lymphoma *in vitro* and *in vivo* [11], [32] and targeting eosinophils using a multivalent glycan ligands of Siglec-8, which induces apoptosis of eosinophils and could be useful in controlling eosinophilic inflammatory responses [33].

Sn/CD169 expression is highly restricted to a subset of resident and activated tissue macrophages [2], [34] making it an attractive receptor for targeting these cells. Recent reports suggest that Sn/CD169-expressing macrophages in the follicle and subcapsular sinus and medulla of lymph nodes play an important role in capturing and processing extracellular antigens and interacting with immune cells to initiate adaptive immune responses [35]–[39]. Thus, these cells are interesting targets for inducing an immune response, and several recent reports demonstrate an enhancement of an immune response by delivering antigens to them using anti-Sn/CD169 antibody [5], [6].

Here we have demonstrated that Sn/CD169 ligand decorated nanoparticles is an efficient alternative to the use of anti-Sn antibody as a means for delivery of antigens to macrophages. 3′-^BPC^NeuAc-liposomes developed in this study is approximately 100 nm in size, which likely allows Sn/CD169 to utilize the clathrin-mediated endocytosis as seen in the case of anti-Sn antibody internalization [5]. A potential advantage of the ligand targeted-liposomes as a delivery platform is their accumulation in lysosomes. Indeed, the sub-cellular localization of the endocytosed ligand-decorated liposomes in lysosomes is in contrast to the subcellular localization of anti-Sn antibody studied by Delputte et al. [5], where the majority of the antibody remained on the surface of the cell, and endocytosed antibody was found only in early endosomes, never in lysosomes. We believe that these results are completely consistent with ours, and reflect the fact that Sn is a recycling receptor, where at anytime, most of the Sn/CD169 is extracellular. Once endocytosed by Sn/CD169, the ligand decorated liposomes and antibody are handled differently. In analogy to what we have found for CD22 [19], we suggest that the ligand-decorated liposomes are released in the acidic endosomes, and then traffic to lysosomes. In contrast, antibody is not efficiently released, and tracks the subcellular localization of Sn/CD169. Thus, as shown by Delputte et al, anti-Sn remains predominately on the outside of the cell, and is found only in early endosomes. The fact that the ligand-targeted liposomes are released and accumulate in endosomal compartments is a potential advantage for the use of this platform for delivery of antigens to macrophages. However, studies comparing several different commercial sources of anti-human Sn and anti-murine Sn showed that some antibodies do not accumulate (Figure 2B), while others slowly accumulate inside the cell, suggesting that release of anti-Sn antibodies in endosomes may be antibody dependent (data not shown).

The idea of targeting antigens to immune cells using receptors that recognize carbohydrate ligands has been investigated by several laboratories [24], [25], [40]–[45]. A good example is dendritic cell-specific intercellular adhesion molecule-3 grabbing nonintegrin (DC-SIGN) which is characterized as the most dendritic cell restricted C-type lectin receptor (CLR). The DC-SIGN functions as an antigen uptake receptor and has a high affinity for both fucose and mannose containing glycans. Recent studies from van Kooyk’s group using a human DC-SIGN transgenic mouse model showed that OVA modified with glycans that specifically target DC-SIGN induces a 7∼10-fold increase of CD4 T cell proliferation and a 2-fold enhancement in proliferation of CD8 T cells [44]. The same group also reported that alteration of glycosylation on the melanoma antigen gp100 with high-mannose structures target DC-SIGN on monocyte-derived dendritic cells, resulting in enhanced antigen presentation to T cells [40]. As macrophages also play an important role in innate and adaptive immune response, several reports have shown that targeting mannosylated antigens to macrophage mannose receptors (MMR) could mediate antigen uptake and presentation by macrophages [24], [40]–[42], [45].

A limitation in the interpretation of results from *in vivo* targeting of antigens to antigen-presenting cells using ligands of glycan-binding proteins is the overlap in the ligand specificity of these receptors, and the degree to which they are expressed on single types of antigen presenting cells. Thus, for example, mannose containing glycans recognized by MMR and the Lewis X structures recognized by DC-SIGN are also recognized by other C-type lectins expressed on dendritic cells and macrophages [46], [47]. Ideally the ligand would be specific for a single receptor, allowing investigations into the efficiency of targeting antigens via that receptor and establishing which cells are contributing to an immune response.

In this report we document a novel platform for delivery of antigens to macrophages comprising liposomal nanoparticles decorated with high affinity glycan ligands of Sn/CD169, a macrophage specific membrane receptor. Antigens targeted to bone marrow derived macrophages induce robust Sn/CD169 dependent activation of antigen specific T cells. Although *in vivo* studies show that the ligand-targeted liposomes are primarily cleared via Sn/CD169 macrophages (Figure S2), and preliminary studies using targeted liposome bearing antigens show dramatically enhanced immune response, the response is not strictly Sn/CD169 dependent (data not shown). We attribute this to the cross-reactivity of the ligand with Siglec-G (Figure 4B) that is expressed on other antigen presenting cells (e.g. dendritic cells and B cells). Thus, more specific ligands are required to perform definitive studies evaluating the potential of Sn/CD169 targeted liposomes *in vivo*.

## Supporting Information

Figure S1 **Confocal microscopy reveals surface localization of Sn.** CHO cells expressing hSn were detected by anti-Sn (green) antibody and were co-stained with antibodies that detect early endosomes (red) or lysosomes (red). The nuclei were visualized by staining cells with DAPI (blue). See Methods S1.
(DOCX)

Figure S2 **Sn-targeted liposomes in the plasma are cleared faster in the wild-type animals than in the Sn−/− mice.** Wild type C57BL/6 (filled symbols) and Sn−/− (open symbols) mice (n = 3) were i.v. injected with naked (circles) or 3′-BPCNeuAc (triangles) liposomes that encapsulate equal amount of doxorubicin. Plasma samples were collected from mice at indicated time points and analyzed for the remaining doxorubicin concentration in the plasma comparing to the initial injection dose. These studies were done as part of a larger study, a portion of which has been published (see References in Methods S1), including the results with the naked liposomes that serve as the control group in this experiment.
(DOCX)

Figure S3 **Sn-targeted liposomes bind to IFN-α stimulated BMM.** (A) Histograms of F4/80 expression on the mature BMM that were stimulated with indicated cytokines followed by staining with FITC-conjugated anti-mouse F4/80 (filled blue) or isotype (light gray) antibodies prior to FACS analysis. Percentages of myeloid-gated F4/80+ BMM are indicated. (B) Cytokine-stimulated BMM were compared for binding of fluorescent 3′-BPCNeuAc liposomes (filled green) and naked liposomes (light gray). Percentages of 3′-BPCNeuAc liposomes bound BMM are indicated.
(DOCX)

Methods S1 **Confocal microscopy and pharmacokinetic study.**
(DOC)
