# Supplementary material for: Antigen Delivery to Macrophages Using Liposomal Nanoparticles Targeting Sialoadhesin/CD169
Source: PLoS One. 2012 Jun 19;7(6):e39039. doi: 10.1371/journal.pone.0039039 (PMC3378521; doi:10.1371/journal.pone.0039039)

**Figure S1. Confocal microscopy reveals surface localization of Sn**. CHO cells expressing hSn were detected by anti-Sn (*green*) antibody and were co-stained with antibodies that detect early endosomes (*red*) or lysosomes (*red)*. The nuclei were visualized by staining cells with DAPI (*blue*)


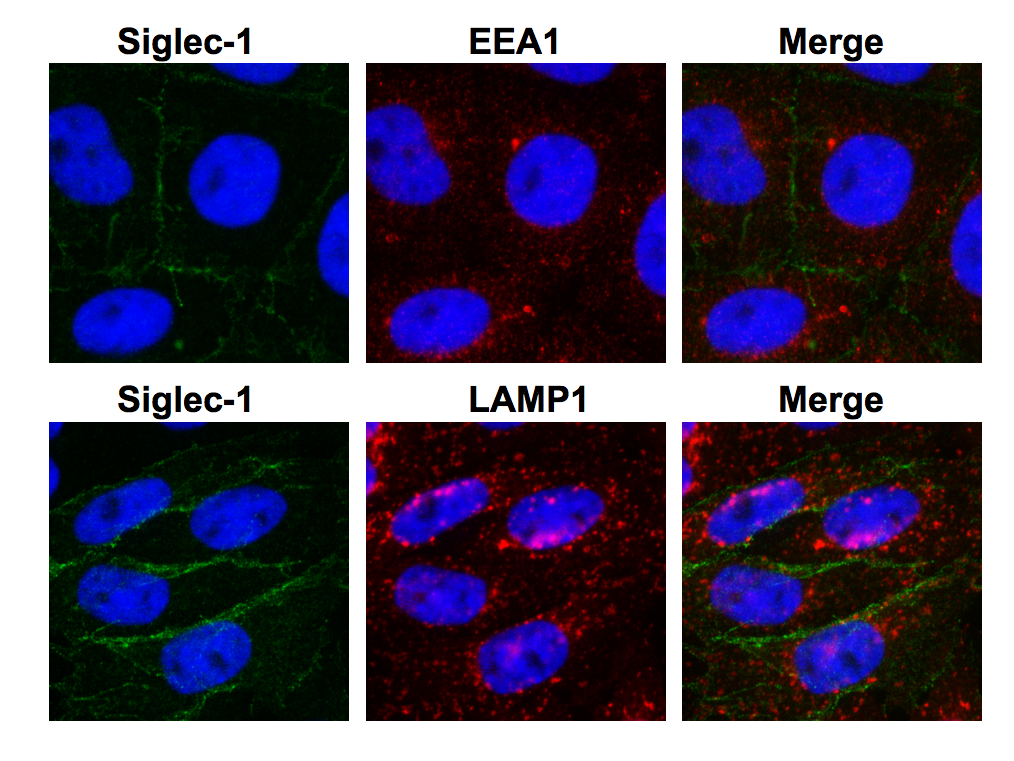

Supplement: Figure S1 — Confocal microscopy reveals surface localization of Sn. CHO cells expressing hSn were detected by anti-Sn (green) antibody and were co-stained with antibodies that detect early endosomes (red) or lysosomes (red). The nuclei were visualized by staining cells with DAPI (blue). See Methods S1. (DOCX) [file pone.0039039.s001.docx]
