# Supplementary material for: Antigen Delivery to Macrophages Using Liposomal Nanoparticles Targeting Sialoadhesin/CD169
Source: PLoS One. 2012 Jun 19;7(6):e39039. doi: 10.1371/journal.pone.0039039 (PMC3378521; doi:10.1371/journal.pone.0039039)

**Figure S2. Sn-targeted liposomes in the plasma are cleared faster in the wild-type animals than in the Sn^-/-^ mice**. Wild type C57BL/6 (filled symbols) and Sn^-/-^ (*open symbols*) mice (n = 3) were i.v. injected with naked (*circles*) or 3′-^BPC^NeuAc (*triangles*) liposomes that encapsulate equal amount of doxorubicin. Plasma samples were collected from mice at indicated time points and analyzed for the remaining doxorubicin concentration in the plasma comparing to the initial injection dose. These studies were done as part of a larger study, a portion of which has been published [see references in Methods S1], including the results with the naked liposomes that serve as the control group in this experiment.


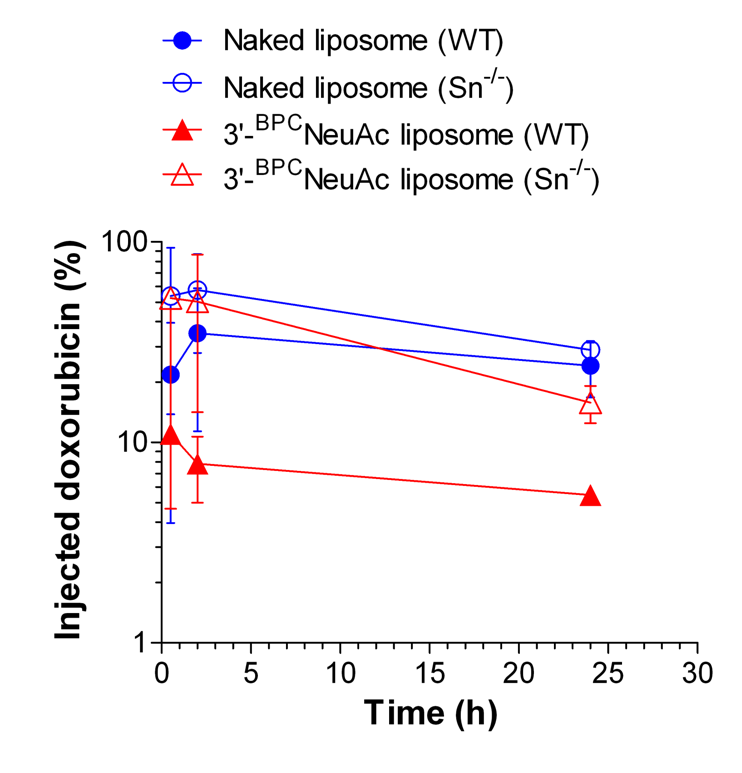

Supplement: Figure S2 — Sn-targeted liposomes in the plasma are cleared faster in the wild-type animals than in the Sn−/− mice. Wild type C57BL/6 (filled symbols) and Sn−/− (open symbols) mice (n = 3) were i.v. injected with naked (circles) or 3′-BPCNeuAc (triangles) liposomes that encapsulate equal amount of doxorubicin. Plasma samples were collected from mice at indicated time points and analyzed for the remaining doxorubicin concentration in the plasma comparing to the initial injection dose. These studies were done as part of a larger study, a portion of which has been published (see References in Methods S1), including the results with the naked liposomes that serve as the control group in this experiment. (DOCX) [file pone.0039039.s002.docx]
