# Supplementary material for: Antigen Delivery to Macrophages Using Liposomal Nanoparticles Targeting Sialoadhesin/CD169
Source: PLoS One. 2012 Jun 19;7(6):e39039. doi: 10.1371/journal.pone.0039039 (PMC3378521; doi:10.1371/journal.pone.0039039)

**Figure S3. Sn-targeted liposomes bind to IFN-α stimulated BMM.** (**A**) Histograms of F4/80 expression on the mature BMM that were stimulated with indicated cytokines followed by staining with FITC-conjugated anti-mouse F4/80 (*filled blue*) or isotype (*light gray*) antibodies prior to FACS analysis. Percentages of myeloid-gated F4/80^+^ BMM are indicated. (**B**) Cytokine-stimulated BMM were compared for binding of fluorescent 3′-^BPC^NeuAc liposomes (*filled green*) and naked liposomes (*light gray*). Percentages of 3′-^BPC^NeuAc liposomes bound BMM are indicated.


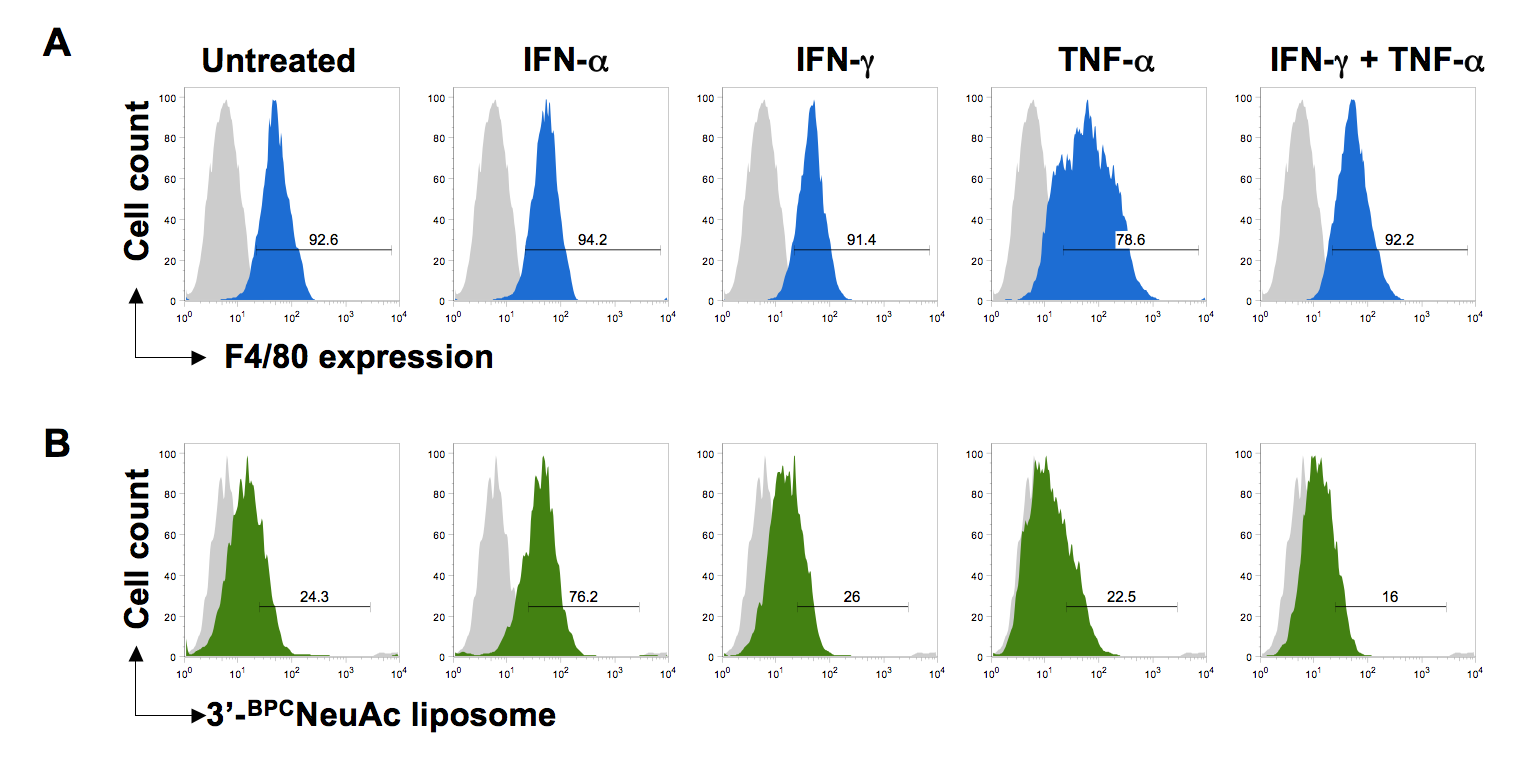

Supplement: Figure S3 — Sn-targeted liposomes bind to IFN-α stimulated BMM. (A) Histograms of F4/80 expression on the mature BMM that were stimulated with indicated cytokines followed by staining with FITC-conjugated anti-mouse F4/80 (filled blue) or isotype (light gray) antibodies prior to FACS analysis. Percentages of myeloid-gated F4/80+ BMM are indicated. (B) Cytokine-stimulated BMM were compared for binding of fluorescent 3′-BPCNeuAc liposomes (filled green) and naked liposomes (light gray). Percentages of 3′-BPCNeuAc liposomes bound BMM are indicated. (DOCX) [file pone.0039039.s003.docx]
