# Supplementary material for: Antigen Delivery to Macrophages Using Liposomal Nanoparticles Targeting Sialoadhesin/CD169
Source: PLoS One. 2012 Jun 19;7(6):e39039. doi: 10.1371/journal.pone.0039039 (PMC3378521; doi:10.1371/journal.pone.0039039)
Supplement: Methods S1 — Confocal microscopy and pharmacokinetic study. (DOC) [file pone.0039039.s004.doc]

**Methods S1.**

Confocal microscopy

CHO cells expressing hSn were plated to a cover slip to achieve a 90% confluence. To analyze Sn localization in the steady state condition, cells were fixed with 3% paraformaldehyde in PBS for 10 min at 25˚C and permeabilized with PBS containing 0.01% saponin and 0.5% BSA for 3 min at 25˚C. Cells stained with anti-human Sn Ab (7D2, Abcam) were co-stained with either anti-EEA1 (ab2900, Abcam) or anti-LAMP1 (ab24170, Abcam) followed by Alexa488 conjugated anti-mouse IgG (Invitrogen) and Alexa555 conjugated anti-rabbit IgG (Invitrogen). Finally the specimens were mounted on a slide using an anti-fade mounting solution (Invitrogen) containing DAPI that stains nuclei. Images were taken using a Bio-Rad Radiance 2100 Rainbow laser scanning confocal microscope and analyzed by ImageJ software.

Pharmacokinetic study

Wild type or Sn^-/-^ mice with C57BL/6 background were intravenously injected with one dose of naked or 3′-^BPC^NeuAc liposomes containing doxorubicin (3 mg/Kg). At 0.5, 2 and 24 hr after liposome injection, a sample of blood (50~100 μl) was collected in an EDTA-coated tube to prevent plasma from clogging. Plasma samples were prepared as described [1] prior to the analysis for the plasma concentration of the extracted doxorubicin using a fluorescence plate reader.

**References:**

1. Chen WC, Completo GC, Sigal DS, Crocker PR, Saven A, et al. (2010) In vivo targeting of B-cell lymphoma with glycan ligands of CD22. Blood 115: 4778-4786.
